# Size Uncertainty in Individual Nanoparticles Measured by Single Particle Inductively Coupled Plasma Mass Spectrometry

**DOI:** 10.3390/nano13131958

**Published:** 2023-06-28

**Authors:** Shuji Yamashita, Shin-ichi Miyashita, Takafumi Hirata

**Affiliations:** 1 National Metrology Institute of Japan (NMIJ), National Institute of Advanced Industrial Science and Technology (AIST), Tsukuba Central 3-9, 1-1-1 Umezono, Tsukuba 305-8563, Ibaraki, Japan; shinichi-miyashita@aist.go.jp; 2 Geochemical Research Centre, The University of Tokyo, Hongo 7-3-1, Bunkyo-ku, Tokyo 113-0033, Japan; dept4706@g.ecc.u-tokyo.ac.jp

**Keywords:** nanoparticle, single particle ICP-MS, size analysis, uncertainty

## Abstract

Single particle inductively coupled plasma mass spectrometry has been used for size measurements of individual nanoparticles (NPs). Here, uncertainties in size analysis based upon two calibration approaches were evaluated: (i) the use of particle size standard and (ii) the use of ion standard solution. For particle size standard approach, the source of uncertainty to determine the target NP diameter was related to the variation in the signal intensities of both target NPs and particle size standard, and the size distribution of the particle size standard. The relative uncertainties of the 50 nm silver NP as the target were 15.0%, 9.9%, and 10.8% when particle size standards of 30 nm, 60 nm, and 100 nm silver NPs were used, respectively. As for the ion standard solution approach, the sources of uncertainty were the concentration of working standard solution, sample flow rate, transport efficiency, slope of calibration curve, and variation in the signal intensity of the ion standard solution and of the target NPs. The relative uncertainties for the 50 nm silver NP were 18.5% for 1 ng/g, 7.6% for 10 ng/g, and 4.7% for 100 ng/g solutions. The lower uncertainty obtained with a high concentration working standard solution is recommended to improve precision on particle size determinations by spICP-MS.

## 1. Introduction

Single particle inductively coupled plasma mass spectrometry (spICP-MS) is a methodology for the characterization of nanoparticles (NPs). Since its introduction by Degueldre [1,2], spICP-MS has become a useful analytical technique for single NPs due to its high sensitivity, elemental selectivity, and high throughput [3,4,5]. Moreover, the analytical capability for size analysis achieved by spICP-MS has revealed that data quality is comparable with those by dynamic light scattering or electron microscopy [6,7].

In the ICP ion source, atoms consisting of NPs are successively atomized and ionized, generating a burst of ions (one ion cloud per particle). Each particle is detected as a pulsed signal (particle event), with a duration of approximately 500 µs. spICP-MS provides the number of atoms (mass) of individual particles from the pulsed signals, and thus, a particle diameter can be calculated from the determined mass. Furthermore, this technique also offers information on the number-based size distribution of particles in the sample. The shape of the size distribution provides insights regarding the state of the NPs in the suspension, such as whether the particles are monodispersed, degraded, or aggregated.

There are several approaches to determine the size of NPs. The intuitive approach is to generate a calibration curve from the signal intensities of measured particles [8,9,10]. This approach requires single or multiple particle size standards, which have the same chemical composition and density as the target NPs. Although this is a useful approach for size calibration, it is restricted by the availability of characterized particle size standards (i.e., particles must be monodispersed and are of known sizes). Another approach is to use a mass flux curve from ion standard solutions and then determine the particle size from the mass of the particle [11]. The density of target NPs is assumed to be equal to the density of the bulk material, and the shape of target NPs is also assumed to be spherical geometry. Size calibration using ion standard solution is commonly applied because particle size standards with the same chemical composition as the target NPs are not required.

At present, the basis and applications of spICP-MS have been described in many articles, recognizing this technique as mature for size analysis [12,13,14,15]. Moreover, several studies have suggested the estimation of the uncertainty in the diameter of single particles [5,7,16,17]. Although many researchers reported particle analysis using spICP-MS, a comprehensive evaluation for the uncertainties of the size analysis for individual particles is still highly required for spICP-MS to become a principal tool for size analysis. In this study, to evaluate the uncertainty of each particle size, two size calibration approaches were conducted: (i) sizing with particle size standard and (ii) sizing with ion standard solution. The uncertainties of the single particle size for each size calibration approach were calculated by evaluating parameters such as signal intensity, working standard concentration, and counting statistics derived from the sample flow rate, and then, the ideal analytical condition for high-precision size analysis was investigated.

## 2. Materials and Methods

### 2.1. Materials

**Figure 1:**
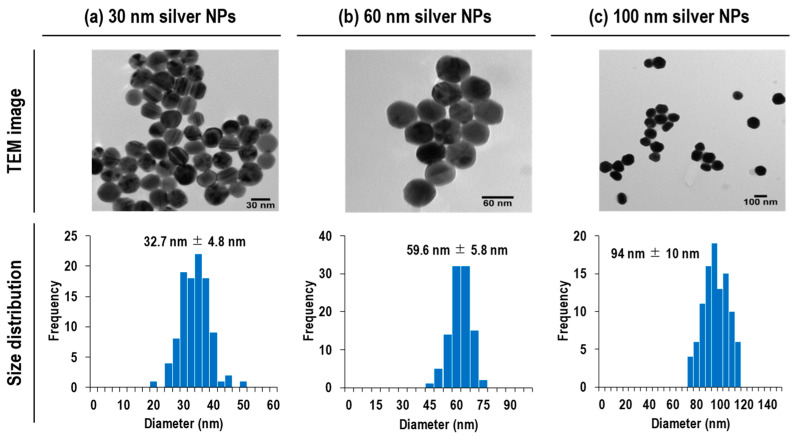
TEM images and size distributions obtained for 30 nm, 60 nm, and 100 nm silver NPs. These data were provided by the manufacturer.

Silver NP suspensions with nominal diameters of 30 nm, 60 nm, and 100 nm were purchased from nanoComposix (San Diego, CA, USA). These silver NPs were citrate-stabilized particles. The reported mean diameter and standard deviation by transmission electron microscopy (TEM, JEOL 1010, Jeol, Tokyo, Japan) of the NPs were 32.7 nm ± 4.8 nm, 59.6 nm ± 5.8 nm, and 94 nm ± 10 nm, respectively. TEM images and size distributions of 30 nm, 60 nm, and 100 nm silver NPs are displayed in Figure 1. The used particle suspensions in this study showed a monodispersed size distribution. These data were provided by the manufacturer.

All silver NP suspensions were diluted in pure water (18.4 MΩ∙cm; Milli-Q Advantage A10, Merck, Rahway, NJ, USA). The number concentrations of the silver NP suspensions were adjusted to be about 25,000 particles/mL. The particle suspensions were prepared before the measurement by spICP-MS to minimize particle dissolution and/or aggregation.

A silver standard stock solution with a mass fraction of 1003 μg/g silver (Kanto Chemical Co., Inc., Tokyo, Japan) was used. Firstly, the standard stock solution was diluted in pure water to generate 1 μg/g standard solution, which was then diluted further to generate three working standard solutions at 1 ng/g, 10 ng/g, and 100 ng/g in 1% HNO_3_. Individual contributions to the uncertainty associated with the concentration of the working standard solutions are summarized in Table 1. The combined standard uncertainty of working standard solutions estimated ranged from 0.45% (1 ng/g) to 0.40% (100 ng/g). The uncertainty of working standard solutions was heavily dependent on the standard stock solution.

### 2.2. Instrumentation

A quadrupole-based ICP-MS (Agilent 8900, Agilent Technologies, Santa Clara, CA, USA), with a conventional MicroMist nebulizer and spray chamber cooled at 2 °C, was used. The sample flow rate was 0.1095 g/min, which was experimentally determined. The instrument was tuned with optimum signal intensity and stability under typical operational settings. Measurements were conducted in the no-gas mode and SP mode at the shortest dwell time of 100 μs. All samples were measured five times for a 60 s period each. The details of the instrumentation and operational settings are listed in Table 2.

### 2.3. Size Analysis

The measurement was conducted based on the TRA (time-resolved analysis) mode. The resulting TRA data were then subjected to the identification of particle events and the calculation of particle sizes from the signal intensity of particle events. In this study, in-house software (NanoQuant v1) was used to identify the particle event and calculate the signal intensity of individual particle events [8,18]. The particle events were identified apart from the background signals using the five times standard deviation criterion. The mean signal intensity and one standard deviation were calculated for the background data set (i.e., Milli-Q water blank), and signals higher than the mean signal intensity plus five times standard deviation were identified as particle events. The other signals were regarded as either electronic noise or dissolved ionic form (non-particulate form). A single particle event was identified as follows: the starting channel was identified by the first data channel of the signal output more than 1 count, and the terminate channel was defined by the first channel of the continuous nil signal output with more than 3 channels. The signal intensity of a single particle event was calculated by integrating the counts between starting and terminal channels.

In this study, two size calibration approaches were tested: (i) sizing with particle size standard [8,18] and (ii) sizing with ion standard solution [11]. The details of the approaches are described below.

(i) Sizing with particle size standard

The calibration factor *F* (nm^3^/counts per particle event) is defined by relating the diameter of the particle size standard and the signal intensity of the particle event calculated from Equation (1),

(1) $$F=\frac{D_{STD\;NP}^{3}}{I_{STD\;NP}}$$

where *D*_STD NP_ is the reported diameter defined by the manufacturer, and *I*_STD NP_ is the signal intensity of a single particle event corresponding to the reported diameter. *I*_STD NP_ is determined as the mode of the signal intensity distribution, fitted with the lognormal distribution measured from the particle size standard. The calibration factor *F*, determined from Equation (1), is then applied to determine the target NP diameter (*D*_target NP_) by relating it to the measured signal intensity of the target NP (*I*_target NP_), as shown in Equation (2).

(2) $$D_{target\;NP}=\sqrt[{3}]{F\times I_{target\;NP}}\;=D_{STD\;NP}\times \sqrt[{3}]{\frac{I_{target\;NP}}{I_{STD\;NP}}}$$

(ii) Sizing with ion standard solution

**Figure 2:**
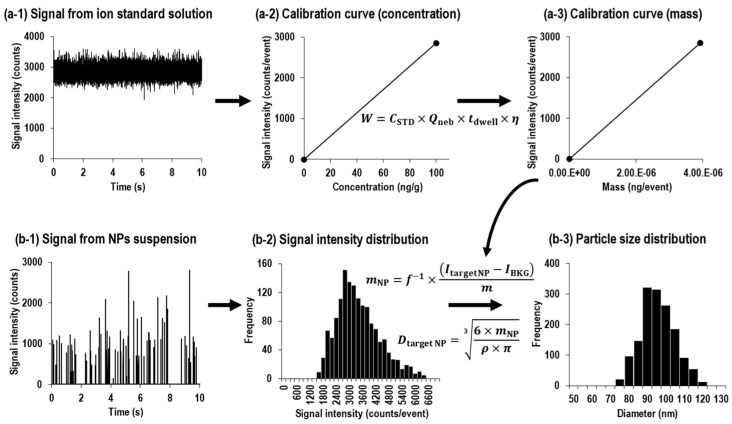
Data processing schematic for the procedures of sizing with ion standard solution by spICP-MS.

**Figure 3:**
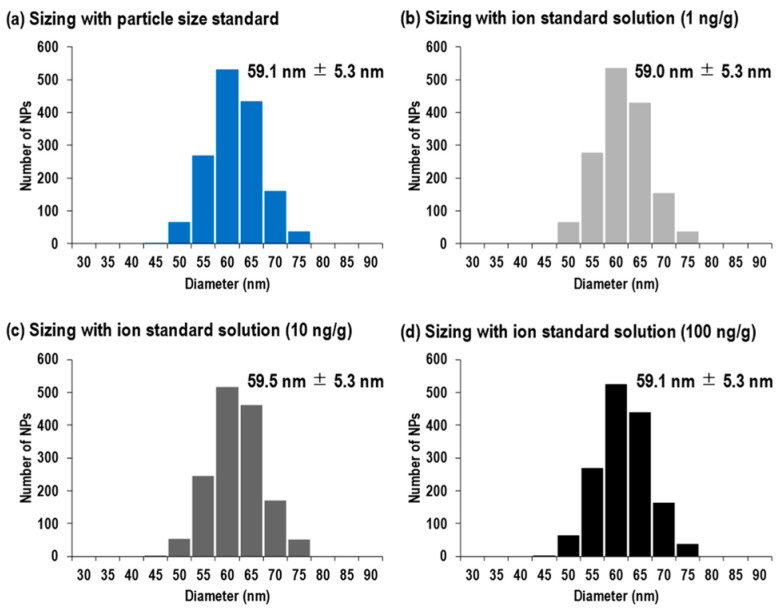
Size distributions of 60 nm silver NPs calibrated by (**a**) 100 nm silver particle size standard, (**b**) 1 ng/g silver ion standard solution, (**c**) 10 ng/g silver ion standard solution, and (**d**) 100 ng/g silver ion standard solution.

Pace et al. developed an approach for size calibration using a mass flux calibration curve from ion standard solutions [11]. Figure 2 shows the data processing schematic for the procedures of sizing with the ion standard solution by spICP-MS. In this approach, a calibration curve is created by relating the concentration of ion standard solutions to signal intensity (Figure 2(a-2)). Then, the concentration of the ion standard solution is converted to mass flux (Figure 2(a-3)) using Equation (3),

(3) $$W=C_{STD}\times Q_{neb}\times t_{dwell}\times \eta \;$$

where *W* is the delivered mass per dwell time (ng), *C*_STD_ is the mass concentration (ng/g), *Q*_neb_ is the sample flow rate (g/s), *t*_dwell_ is the dwell time (s), and *η* is the transport efficiency (%). Mass concentration, sample flow rate, dwell time, and transport efficiency can be obtained by experimental determination. The transport efficiency is defined as the ratio of the amount of analyte entering the plasma to the amount of analyte aspirated. The transport efficiency depends on various operational parameters such as gas flow, sample viscosity, and sample flow rate [19,20,21]. Hence, regular evaluation of transport efficiency is required to obtain accurate size information using the ion standard solution approach.

In order to determine the transport efficiency, three methods are suggested and examined by Pace et al. [11]: (a) waste collection method, (b) particle frequency method, and (c) particle size method. Waste collection method uses solution volume. The total volume of sample introduced to the ICP is calculated by subtracting the spray chamber waste volume collected from the nebulizer sample uptake volume [22]. The transport efficiency is determined by comparing the waste volume with the sample uptake volume. Although the waste collection method is the simplest measurement, it is the least accurate due to evaporation and weighing uncertainties [20]. The particle number method uses particle number concentration to determine the transport efficiency. The transport efficiency is determined by the ratio of the measured number of particle events to the theoretical number of particle events based on the known particle number concentration and sample flow rate. This approach requires accurate particle number concentration. Lastly, the particle size method uses the ratio of ion standard solution sensitivity and particle size standard sensitivity to determine the transport efficiency. The signal intensity obtained from an ion standard solution is dependent on the transport efficiency, while the signal intensity derived from a particle is not. Hence, the ratio of the ion standard solution sensitivity (counts per ng of analyte delivered to the nebulizer) to the particle sensitivity (counts per ng of analyte in a single particle) is equal to the transport efficiency. In this study, the particle size method was applied to determine the transport efficiency.

Then, the signal intensity of each particle event (Figure 2(b-2)) is substituted into the resulting mass flux calibration curve. The obtained signal intensities are converted to the mass of the corresponding particle using Equation (4),

(4) $$m_{NP}=f^{-1}\times \frac{\left(I_{target\;NP}-I_{BKG}\right)}{m}$$

where *m*_NP_ is the mass of the particle, *f* is the mass fraction (the fraction of the particle mass due to the analyte element), *I*_target NP_ is the signal intensity of the particle event, *I*_BKG_ is the signal intensity of the background, and *m* is the slope of the mass flux calibration curve. The resulting *m*_NP_ is converted to diameter (*D*_target NP_) using Equation (5), assuming a spherical geometry.

(5) $$D_{target\;NP}=\sqrt[{3}]{\frac{6\times m_{NP}}{\rho \times \pi }}$$

Here, *ρ* is the particle density, which is assumed to be equal to the bulk density of the targeted material, similar to many previous studies.

### 2.4. Uncertainty Analysis

In order to evaluate the uncertainty of the single particle diameter, we followed the guidance in the “Guide to the Expression of Uncertainty in Measurement” (GUM). The sources of uncertainty and the relative uncertainty of each source were investigated. The contributions of uncertainty sources in this study were mainly evaluated by type A methods, i.e., the degree of uncertainty was quantified by the standard deviation of replicate measurements and counting statistics derived from the signal intensity. Then, the contributions of the uncertainty to the particle size were investigated. The combined standard uncertainty was estimated by the square root of the sum of the squares of the individual relative uncertainties.

## 3. Results and Discussion

### 3.1. Comparison of Size Distributions between Two Size Calibration Approaches

Before evaluating the uncertainty of the single particle diameter, the reliability of the size distributions of 60 nm silver NPs defined by the two size calibration approaches was investigated. Note that both size calibration approaches were applied to the same 60 nm silver NP data, measured from 1500 particles. In this experiment, silver NPs with a nominal diameter of 100 nm (94 nm ± 10 nm) were used as the particle size standard, giving *D*_STD NP_ and *I*_STD NP_ as 94 nm and 3313 counts/event, respectively. By substituting these values into Equation (1), the calibration factor *F* was obtained as 251 nm^3^/counts per particle event.

Figure 3a shows the size distribution of the 60 nm silver NPs, calibrated by using silver NPs with a nominal diameter of 100 nm as the particle size standard. This size distribution was obtained by the calculations from Equation (2). The resulting size distribution was monodispersed, with a mean diameter and its one standard deviation of 59.1 nm ± 5.3 nm. On the other hand, Figure 3b–d illustrate the size distributions calibrated by working standard solutions of (b) 1 ng/g, (c) 10 ng/g, and (d) 100 ng/g silver standard solutions. The mean diameters and their standard deviations were 59.0 nm ± 5.3 nm for 1 ng/g, 59.5 nm ± 5.3 nm for 10 ng/g, and 59.1 nm ± 5.3 nm for 100 ng/g silver standard solutions. These values indicated that there was no significant difference between the size distributions, regardless of the difference in the silver concentration of the standard solutions. Moreover, the size distributions obtained by both size calibration approaches were in good agreement with the reported value from the manufacturer (i.e., 59.6 nm ± 5.8 nm for TEM) within analytical uncertainty, thus demonstrating that spICP-MS is a reliable analytical technique for size distribution analysis.

### 3.2. Uncertainty for Sizing with Particle Size Standard

In the case of sizing with the particle size standard, the diameter of target NP (*D*_target NP_) is obtained using Equation (2). Based on Equation (2), the uncertainty of *D*_target NP_ depends on *I*_STD NP_, *I*_target NP_, and *D*_STD NP_. Hence, the relative uncertainty of *D*_target NP_ can be expressed as Equation (6) based on the basic rules of the propagation of uncertainty.

(6) $$\frac{u(D_{target\;NP})}{D_{target\;NP}}=\sqrt{{\left(\frac{u\left(D_{STD\;NP}\right)}{D_{STD\;NP}}\right)}^{2}+{\left\{\frac{1}{3}\left(\frac{u\left(I_{STD\;NP}\right)}{I_{STD\;NP}}\right)\right\}}^{2}+{\left\{\frac{1}{3}\left(\frac{u\left(I_{target\;NP}\right)}{I_{target\;NP}}\right)\right\}}^{2}}\;=\sqrt{{\left(\frac{u\left(D_{STD\;NP}\right)}{D_{STD\;NP}}\right)}^{2}+{\frac{1}{9}\left(\frac{u(I_{STD\;NP})}{I_{STD\;NP}}\right)}^{2}+\frac{1}{9}{\left(\frac{u(I_{target\;NP})}{I_{target\;NP}}\right)}^{2}}$$

The uncertainty of *I*_STD NP_ or *I*_target NP_ is attributed to counting statistics, in which the uncertainty is given by

(7) $$u\left(I_{STD\;NP\;or\;target\;NP}\right)=\sqrt{I_{STD\;NP\;or\;target\;NP}\;}$$

From Equation (7), the relative uncertainty of *I*_STD NP_ or *I*_target NP_ can be described as Equation (8),

(8) $$\frac{u\left(I_{STD\;NP\;or\;target\;NP}\right)}{I_{STD\;NP\;or\;target\;NP}}=\frac{\sqrt{I_{STD\;NP\;or\;target\;NP}}}{I_{STD\;NP\;or\;target\;NP}}$$

From Equation (2), the signal intensities (*I*_target NP_) corresponding to 10 nm and 100 nm were calculated based on the correction factor *F* (i.e., 251 nm^3^/counts per particle event), resulting in approximately 4 counts/particle event and 3989 counts/particle event, respectively. Based on Equation (8), the relative uncertainties of *I*_target NP_ were 50% for 10 nm and 1.6% for 100 nm. Similarly, the relative uncertainty of *I*_STD NP_ was 1.7% (*I*_STD NP_: 3313 counts/event).

**Figure 4:**
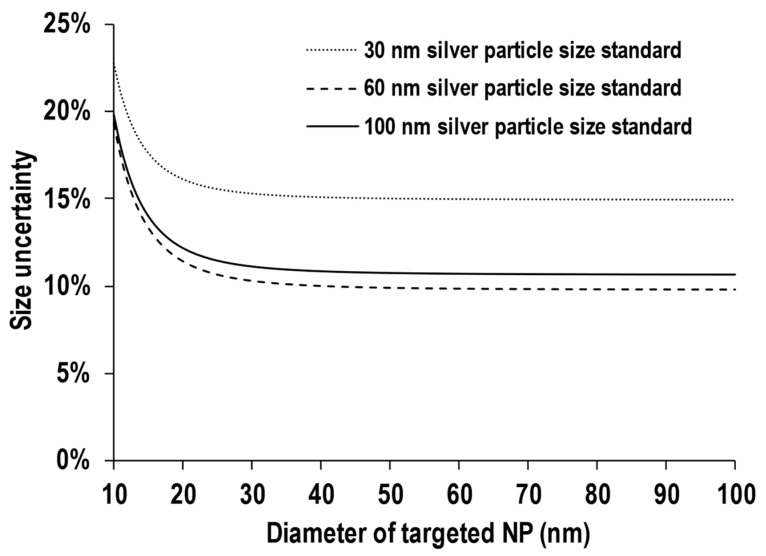
Relative size uncertainty plotted against the diameter of targeted NPs using 30 nm silver NPs, 60 nm silver NPs, and 100 nm silver NPs as the particle size standard.

Figure 4 shows the graph of the relative size uncertainty plotted against the diameter of target NPs using 30 nm, 60 nm, and 100 nm silver NPs as particle size standards. The lines in Figure 4 were calculated based on Equation (6). Due to a larger value of *I*_target NP_ obtained when the size of *D*_target NP_ increased, the relative uncertainty in *D*_target NP_ decreased when *D*_target NP_ is larger. This trend was similar even for particle size standards of different sizes. Since a larger value of *I*_STD NP_ can be obtained from larger particles, one might consider the use of large-sized particle size standards as the most important parameter for high-precision analysis. However, as we can see in Figure 4, the 60 nm silver particle size standard displayed the smallest relative size uncertainty, rather than 100 nm. In addition, relative size uncertainty remains almost unchanged after 40 nm, even when larger target NPs were measured. Other than *I*_target NP_ and *I*_STD NP_, the uncertainty in the size of the particle size standard must also be considered. Based on the reported values of TEM from the manufacturer, the relative uncertainties of *D*_STD NP_ were 14.7% for 30 nm silver NPs, 9.7% for 60 nm silver NPs, and 10.6% for 100 nm silver NPs. From Equation (6), the relative uncertainty obtained from the particle size standard approach is largely dominated by the degree of uncertainty of *D*_STD NP_. Therefore, the use of a particle size standard with a narrower size distribution is ideal to achieve high-precision analysis for individual particle size.

### 3.3. Uncertainty for Sizing with Ion Standard Solution

In the case of sizing with ion standard solution, the sources of uncertainty associated to determine the diameter of the target NP are related to the concentration of the working standard solution (*C*_STD_), sample flow rate (*Q*_neb_), transport efficiency (*η*), slope of the calibration curve (*m*), signal intensity of the working standard solution (*I*_soln_), and signal intensity of target NPs (*I*_target NP_). Each resulting relative uncertainty is listed in Table 3. Based on the sources of uncertainty associated to the determination of the diameter of the target NP, the relative uncertainty of *D*_target NP_ is given by

(9) $$\frac{u(D_{target\;NP})}{D_{target\;NP}}=\sqrt{{\left(\frac{u(C_{STD})}{C_{STD}}\right)}^{2}+{\left(\frac{u(Q_{neb})}{Q_{neb}}\right)}^{2}+{\left(\frac{u(\eta )}{\eta }\right)}^{2}+{\left(\frac{u(m)}{m}\right)}^{2}+{\left(\frac{u(I_{soln})}{I_{soln}}\right)}^{2}+{\left(\frac{u(I_{target\;NP})}{I_{target\;NP}}\right)}^{2}}$$

**Figure 5:**
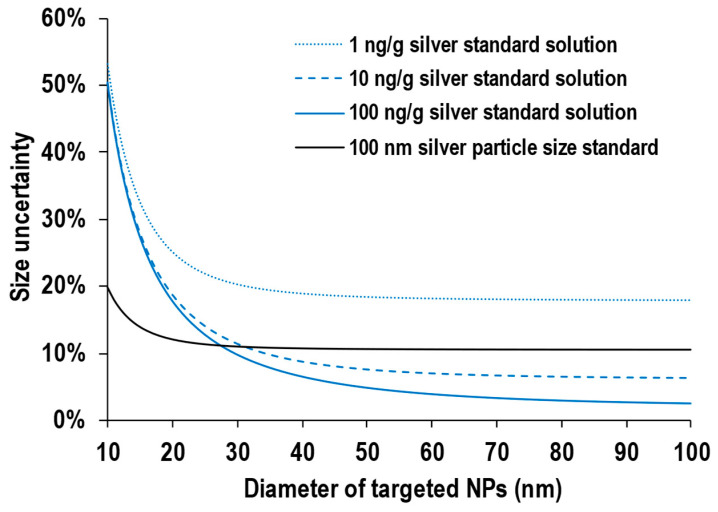
Relative size uncertainty plotted against the diameter of targeted NPs using 1 ng/g silver ion standard solution, 10 ng/g silver ion standard solution, and 100 ng/g silver ion standard solution as the standard. The resulting relative size uncertainty plotted against the diameter of targeted NPs using 100 nm silver NPs as the particle size standard is also shown.

Figure 5 shows the relative size uncertainty plotted against the diameter of target NPs using 1 ng/g, 10 ng/g, and 100 ng/g silver standard solutions as the working standard solutions. These lines in Figure 5 were calculated based on Equation (9). Similar to the resulting relative size uncertainty determined by the particle size standard approach, the relative uncertainty of *D*_target NP_ decreased with increasing *D*_target NP_, regardless of the concentration of the working standard solution. This is because the value of *I*_target NP_ is larger for a larger *D*_target NP_, leading to a smaller relative uncertainty in *I*_target NP_. More importantly, the relative uncertainty of *D*_target NP_ decreased with a higher concentration of the working standard solution (e.g., relative uncertainties in size of 100 nm were 18% for 1 ng/g, 6.3% for 10 ng/g, and 2.6% for 100 ng/g). The higher the concentration of the working standard solution, the higher the signal intensity obtained, and thus, the uncertainty of *I*_soln_ becomes smaller. However, it should be noted that pulse-analog switching will occur at the detection system when the measurement of the higher concentration of the working standard solution exceeds 10^6^ cps. Although the signal intensity obtained from the 100 ng/g working standard solution was 3 × 10^7^ cps in this study, pulse-analog adjustment was conducted before the analysis. Hence, the same linear response over a wide range of signal intensity was exhibited with the pulse counting mode and analog mode.

The results obtained in this study also indicated that the uncertainty of the individual particle size depends on the signal intensity. Therefore, in order to achieve high-precision size analysis for single particles, magnetic sector-based ICP-MS should be applied due to their higher sensitivity compared with quadrupole-based ICP-MS, which is used in this study, while also improving instrumental sensitivity through new developments in the hardware.

In order to compare the degree of the relative size uncertainty between the use of particle size standard and ion standard solution, the relative uncertainty of *D*_target NP_ obtained by 100 nm silver NPs as a particle standard (black line) is also illustrated in Figure 5. It is noted that this line was calculated based on Equation (6). For particles ranging from 10 nm to 30 nm, the relative size uncertainty of the target NP was smaller when sizing with the 100 nm particle size standard. Since the relative uncertainty of *I*_target NP_ and *I*_STD NP_ determined by the particle size standard approach is multiplied by 1/9 (refer to Equation (6)), the effect of the signal intensity derived from the target NP (*I*_target NP_) and particle size standard (*I*_STD NP_) on the relative size uncertainty of the target NP is small. Hence, higher-precision data can be obtained when sizing with the 100 nm particle size standard.

As for particles with size larger than 30 nm, the particle size standard approach is affected by the uncertainty of *D*_STD NP_ (i.e., the size distribution of the particle size standard). Hence, as shown in Figure 5, the uncertainty in sizes of more than 30 nm is limited to approximately 10% when using the particle size standard approach. On the other hand, the uncertainty of *D*_target NP_, obtained by the ion standard solution approach, mainly depends on *I*_soln_. The higher the concentration of the working standard solution, the higher the signal intensity obtained, and thus, the uncertainty of *I*_soln_ decreased. The uncertainty of *I*_soln_ was up to 2% when using the 100 ng/g working standard solution. Hence, higher-precision data can be obtained when sizing with a higher concentration working standard solution than when sizing with the particle size standard.

Based on these results, in order to achieve size analysis with the smallest possible size uncertainty, the size calibration approach using the particle size standard is recommended when the target NPs are smaller than 30 nm, while a higher concentration working standard solution is recommended when the target NPs are larger than 30 nm.

## 4. Conclusions

The size distribution of 60 nm silver NPs that was defined by sizing with 100 nm silver NPs as the particle size standard was comparable with the size distributions defined by sizing with an ion standard solution. In addition, the size distributions calibrated by 1 ng/g, 10 ng/g, and 100 ng/g silver working standard solutions were in good agreement within analytical uncertainty. From these results, we concluded that regardless of the concentration of the working standard solution, reliable size distribution data can be obtained by sizing with the ion standard solution approach.

The focus of this study was on the evaluation of spICP-MS for the uncertainty of the single NP diameter. The relative uncertainty of the target NP diameter defined by sizing with the particle size standard is decreased with an increasing diameter of target NPs regardless of the size of the particle size standard. However, the relative uncertainty of the target NP diameter was limited by the size distribution of the particle size standard, and thus, the use of a particle size standard with a narrower size distribution is ideal to achieve high-precision analysis for individual particle size.

As for sizing with ion standard solution, the relative uncertainty of the target NP diameter decreased with an increasing target NP diameter regardless of the concentration of working standard solutions. Moreover, it was revealed that the precision of the target NP diameter was improved using a working standard solution with high concentration due to the decrease in the counting statistics derived from signal intensity. However, it should be noted that if the signal intensity exceeds 10^6^ cps when a high concentration working standard solution is measured, pulse-analog adjustment must be conducted to make sure that linearity is maintained across a wide range. The results obtained in this study demonstrated that the evaluation of spICP-MS measurement for the uncertainty of the single particle diameter will contribute to the establishment of spICP-MS as a reliable analytical technique for NP size analysis.

Recently, Kaynarova et al. offered an estimation for the size uncertainty of a single silver NP measured by spICP-MS [17]. They investigated the factors influencing the contribution of the signal noise to the size uncertainty of a single silver NP. Similar to our results, the size uncertainty obtained in this previous study indicated that, as the particle size decreases, the uncertainty of the estimated diameter increases. However, the size accuracy obtained in our study was higher because of better instrumental sensitivity and experimental conditions.

The results obtained in this study indicated that the uncertainty of individual particle size was mainly affected by signal intensity, and thus, ICP-MS with higher sensitivity is ideal. Magnetic sector-based ICP-MS has the highest instrumental sensitivity, followed by quadrupole-based ICP-MS and time-of-flight-based ICP-MS. The use of the magnetic sector-based ICP-MS as well as improving instrumental sensitivity through new developments such as the sample introduction system and mass analyzer will be required to obtain reliable and high-precision size data for single particles.

## Figures and Tables

**Table 1:** Uncertainty associated with the preparation of calibration standard solutions by weighing.

Description	Value	Standard Uncertainty	Relative Uncertainty
Concentration of silver standard stock solution	1003 μg/g	3.5 μg/g ^1^	0.35%
Weight of standard stock solution	0.1 g (1 μg/g)	0.0002 g ^2^	0.2%
Weight of 1 μg/g standard solution	0.1 g (1 ng/g), 1.0 g (10 ng/g), 10 g (100 ng/g)	0.0002 g ^2^	0.2% (1 ng/g), 0.02% (10 ng/g), 0.002% (100 ng/g)
Weight of dilution	100 g	0.0002 g ^2^	0.0002%
Combined standard uncertainty	–	–	0.45% (1 ng/g), 0.40% (10 ng/g), 0.40% (100 ng/g)

^1^ Certified by manufacturers. ^2^ Mass uncertainties for an analytical balance, ±0.0002 g.

**Table 2:** Instrumentation and operational settings.

**Instrumental Parameters**	
ICP-MS Instrument	Agilent 8900
RF power	1550 W
Argon gas flow rate	
Plasma gas	15.0 L/min
Auxiliary gas	0.90 L/min
Nebulizer gas	1.20 L/min
Nebulizer type	MicroMist nebulizer (for 8900 Standard and #100)
Spray chamber type	Double-pass
Spray chamber temperature	2 °C
Gas mode	No gas
**Data acquisition parameters**	
Acquisition mode	SQ
Dwell time	100 μs
Settling time	-
Monitored isotopes	^107^Ag

**Table 3:** Relative uncertainty associated with the determination of diameter of each NP using ion standard solution.

Source	Relative Uncertainty
Concentration of working standard solutions (*C*_std_) ^1^	0.45% (1 ng/g), 0.40% (10 ng/g), 0.40% (100 ng/g)
Sample flow rate (*Q*_neb_) ^2^	0.69%
Transport efficiency (*η*) ^2,3^	0.34%
Slope of calibration curve (*m*) ^2^	0.13% (1 ng/g), 0.092% (10 ng/g), 0.045% (100 ng/g)
Signal intensity of working standard solutions (*I*_soln_) ^4^	18% (1 ng/g), 6.0% (10 ng/g), 1.9% (100 ng/g)
Signal intensity of particles (*I*_NPs_) ^4^	50% (4 counts/event = ca. 10 nm) − 1.6% (4000 counts/event = ca. 100 nm)

^1^ Calculation procedure. ^2^ Experimentally determined. ^3^ Calculation procedure based on particle size method (Pace et al., 2011) [11]. ^4^ Counting statistics.

## Data Availability

The data presented in this study are available on request from the corresponding author. The data are not publicly available due to privacy reasons.
